# A dataset on the experimental study of online and offline communication with digital and biomarkers

**DOI:** 10.1038/s41597-026-07382-1

**Published:** 2026-05-14

**Authors:** Elina Tsigeman, Larisa Mararitsa, Olga Lopatina, Ailar Avliyakulyeva, Ekaterina Kindyakova, Olessia Koltsova

**Affiliations:** 1 HSE University, The Social and Cognitive Informatics Laboratory, Saint-Petersburg, Russia; 2 Krasnoyarsk State Medical University named after Prof. V.F. Voino-Yasenetsky, Social Neuroscience Laboratory, Krasnoyarsk, Russia

**Keywords:** Human behaviour, Social behaviour

## Abstract

The rise of videoconferencing (VC) technologies has transformed how individuals collaborate and interact across professional and personal contexts. However, empirical studies comparing VC and face-to-face (FtF) interactions remain fragmented, partly due to a lack of open, multimodal datasets capturing both modalities with rich behavioural, physiological, and self-report measures. To address this gap, we introduce the dataset, comprising approximately 180 hours of audio-visual recordings of unacquainted dyads engaging in structured and creative collaborative tasks under controlled laboratory conditions. Participants were randomly assigned to VC or FtF interaction. The dataset includes six salivary oxytocin measurements, self-reports on affect, personality traits, relevant attitudes, communication outcomes, and a repeated sustained attention task. Behavioural recordings from frontal and side camera views are available for most dyads, with individual-level data for 127–131 participants. The dataset enables research into social bonding, cooperation, behavioural and physiological synchrony, and broader communication dynamics, filling a critical gap in resources for comparative communication studies.

## Background & Summary

The widespread adoption of videoconferencing (VC) technologies in recent years, further accelerated by the COVID-19 pandemic, has led to their normalisation across diverse domains, including medical consultations^1^, psychotherapy^2^, education^3^, legal proceedings^4^, employment interviews^5^, and behavioural research^6^. This shift has prompted renewed interest in how VC affects interpersonal communication, both in the dynamics of moment-to-moment interaction and in broader psychological and practical outcomes.

Theoretical perspectives offer differing predictions regarding these effects. On one hand, frameworks such as social presence theory^7^, media richness theory^8^, and the social identity model of deindividuation^9^ suggest that VC — characterised by reduced access to non-verbal cues compared to face-to-face (FtF) interaction — may attenuate intimacy, identifiability, sensitivity to interpersonal similarity, and mutual understanding. On the other hand, models such as social information processing theory^10^, media compensation theory^11^, and the hyperpersonal model^12^ propose that communicators may adapt their behaviour to overcome these limitations, or even leverage the unique affordances of VC for self-presentation and relationship building.

Despite the range of theoretical predictions, empirical findings remain fragmented, as suggested by existing reviews^13^. Some studies report lower creativity^14^, diminished behavioural^15^ and neural synchrony^16^, reduced interpersonal attraction^17^, increased fatigue^18,19^, and weakened conversational turn-taking and cooperation^19^ in VC relative to FtF interaction. Others find comparable or even enhanced outcomes in VC settings for certain types of collaboration, such as idea selection^14^.

A major challenge in reconciling existing findings is the limited availability of open, multimodal datasets that allow systematic, comparative investigation of VC and FtF interactions. Notably, none of the aforementioned empirical studies provides public access to their datasets. To the best of our knowledge, no open dataset contains data for both VC and FtF conditions. For example, SEWA DB^20^ and NoXI^21^, while offering exceptionally rich annotations, feature only screen-mediated dyadic interactions. In contrast, ECOLANG^22^, with equally detailed annotation, and UDIVA^23^, which combines audio-visual recordings with multiple physiological sensors, focus exclusively on FtF interactions. Moreover, most existing resources have additional limitations: for instance, they may lack physiological measures (e.g., SEWA DB^20^, NoXI^21^), cooperative tasks (e.g., K-EmoCon^24^), oral communication (e.g., PhotoChat^25^), or may be based on scripted or artificial interactions (e.g., MELD^26^). To our knowledge, no current dataset combines naturalistic dyadic cooperative interaction across modalities with behavioural recordings, repeated biological sampling, and rich self-report data.

**Fig. 1:**
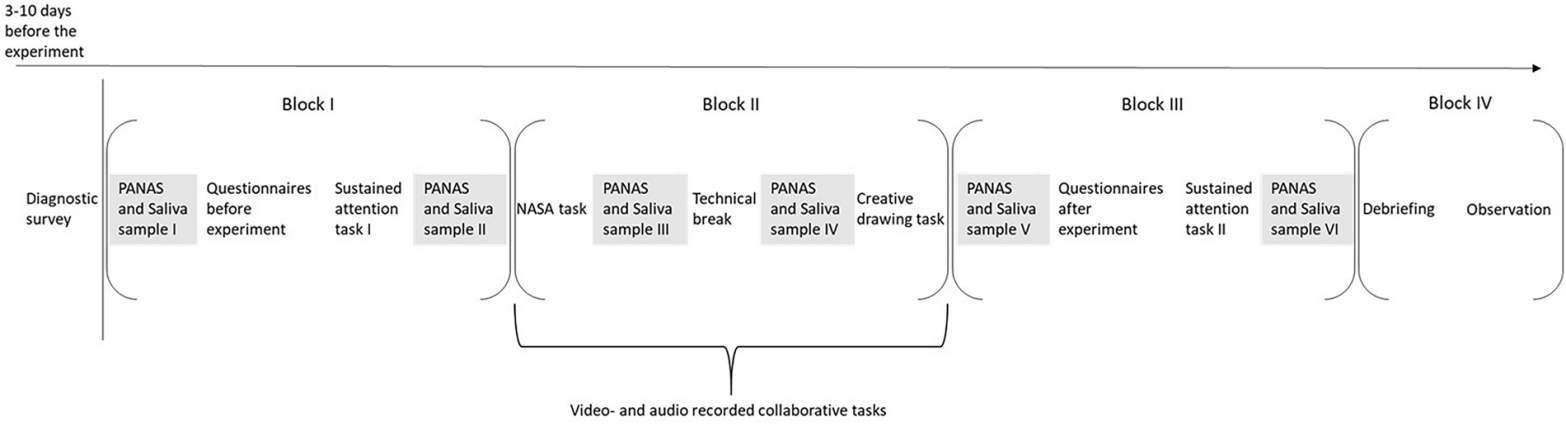
A schematic overview of the experimental procedure.

To address this gap, we introduce a multimodal dataset of dyadic communication across both face-to-face and virtual modalities, with a visualisation of the experimental workflow provided in Fig. 1. The dataset comprises approximately 180 hours of audio- and video-recorded interactions between strangers engaged in structured and creative collaborative tasks in laboratory settings. Each dyad was randomly assigned to communicate either via VC or in person. All interactions and surveys were conducted in Russian, with participants being native speakers.

Individual-level data include three major components: (1) six repeated measurements of saliva oxytocin, a potential biomarker of social bonding, collected across the course of the session; (2) two measurements of sustained attention task; and (3) diverse self-report assessments, comprising (a) six repeated measures of positive and negative affect, (b) pre-interaction assessments of psychological traits (Big Five personality traits, Zoom fatigue, attitudes toward virtual meetings, social concerns, and close relationship experience type), and (c) post-interaction measures of perceived partner attraction and social presence, communication satisfaction, and attributional confidence.

While the full dataset contains 65 dyads, individual data are publicly available for between 127 and 131 participants, depending on the measure. Dyadic audio-visual recordings are available for 55 pairs (frontal camera view).

The dataset is designed to support a broad range of research goals, including investigations into social bonding, communication effectiveness, physiological and behavioural synchrony, and AI-based modelling of social signals. With the data collected under controlled laboratory conditions, the design captures ecologically relevant, unscripted conversations between unacquainted individuals. Some limitations apply: not all dyads are available in both video views, and while the video quality is sufficient for analysing posture and turn-taking behaviour, it is less suited to fine-grained emotion recognition. A notable drawback of this study is the lack of uniformity in the timing of saliva sampling. Samples were collected immediately before and after each task. However, no consideration was given to the exact amount of time that elapsed between these points. Nonetheless, the dataset provides a rare combination of behavioural, physiological, and subjective data across matched online and offline interactions, offering new opportunities for comparative research on human communication

## Methods

### Participants

Participants were native Russian speakers aged between 18 and 25 years recruited from university student samples in two cities (St. Petersburg and Krasnoyarsk) through online invitations. Exclusion criteria for participants comprised medication use (e.g., anxiolytics), illness (hormonal and infectious) and pregnancy.

One hundred and fifty participants formed seventy-five same-sex dyads of strangers. Out of them, 131 participants agreed to share their data publicly. The final public dataset thus contains individual data for these 131 participants and dyadic data for 65 dyads, thus completely excluding recordings and transcripts from those dyads in which at least one member declined sharing their data. Details of the demographics of the study participants, who allowed data publication, are shown in Table 1.

**Table 1:** Demographics of study participants in study conditions.

	FtF	VC
**N of females**	36	39
**N of males**	38	18
**Total N**	74	57
**Age (SD)**	20.76 (1.93)	20.18 (1.82)
**Age range**	18–24	18–25

### Procedure

The study consisted of two phases: a screening survey and an experimental part. All task instructions, communication tasks, and survey instruments were administered in Russian. The first phase was conducted online 3–10 days before the experiment and took no more than 15 minutes to complete. During this stage, participants received information about the study and completed a screening survey assessing sociodemographic (age, binary gender), medical history (medications, illnesses), video conferencing use experience, and social anxiety. They also provided informed consent for data processing and their contact details.

Following the survey, a coordinator assessed information on age, sex, medication use, and medical conditions that served as exclusion criteria for participation in the experiment. Eligible individuals were contacted and invited to participate in the experimental part of the study. To ensure that a dyad consisted of strangers, participants were asked to provide their photographs. These photographs were shared (with the participant’s consent) with a same-sex candidate partner. Participants then reported whether their potential partner was familiar to them. If one person in the dyad reported familiarity with the potential matching partner, another candidate was provided until both participants in the dyad confirmed that they did not know each other. Finally, a date and time for the experimental session was agreed with the participants.

The second phase of the study was a 2–2.5-hour laboratory experiment comprising four blocks (Fig. 1). The experiment was conducted by a blinded experimenter unaware of the study hypotheses. This person accompanied participants during questionnaire completion but was absent during joint task performance. Instead, the experimenter monitored the procedure via audio-only videoconference, which broadcasted task instructions and timing. All rooms in which the experiment was conducted were isolated.

Upon arrival to the experimental site, participants completed a written informed consent form for experiment participation and were given instructions on the procedure of the experiment, but not the details of the purpose of the experiment. Next, they were randomly assigned to one of the two conditions — Videoconference or Face-to-Face communication — with identical block structures. The procedure ensured participants had no in-person contact prior to the experiment, during the instruction stage and in blocks I and III of the experiment, thus limiting their co-presence to blocks II (collaborative tasks) and IV (debriefing).

Block I of the experiment lasted approximately 20 minutes and consisted of tests and questionnaires. Participants completed a questionnaire on positive and negative affect and provided a baseline saliva sample (Assessment 1). The first saliva sample was collected under the supervision of a medical assistant; subsequent samples were self-collected.

Next, participants filled in several questionnaires (see section Block I for details), and performed a sustained attention task. At the end of Block I, participants again completed a questionnaire on positive and negative affect and provided a second saliva sample (Assessment 2).

Block II, which lasted for 41 minutes, comprised three sequential activities conducted either face-to-face or online (NASA Exercise: Survival on the Moon, unrestricted behaviour during contrived technical break and Creative drawing task; for full description see Block II section (Collaborative tasks). Participants filled in a questionnaire on positive and negative affect and submitted saliva samples after each task (Assessments 3–5).

Members of dyads assigned to the VC communication condition sat in two separate rooms. Each participant was seated at a table in front of a laptop at the distance of 50–70 cm. Differences in distance were due to individual preferences in comfort and lack of rigid head fixation. Participants interacted via videoconferencing using Zoom (Zoom Video Communications). The VC software was set to full screen with no self-view windows.

Members of dyads assigned to the FtF communication condition were seated in the same room. These participants sat on chairs facing each other while communicating and performing tasks. Each participant in FtF condition also had a laptop on the table in front of them to get the text instructions on the screen.

In both conditions, audio and video recordings of the participants were collected using two portable video cameras and two built-in laptop cameras to capture voice, front and side views of the participants. The portable video cameras were positioned at a distance of approximately 1 meter and aimed at each participant’s face from the side. Participants were encouraged to adopt a comfortable posture, but were asked to keep their face in the center of the camera.

Block III of the experiment that took around 20 minutes included the second performance of the sustained attention task and the completion of questionnaires about the quality of social contact with the partner. After that, participants filled in the positive and negative affect questionnaire and provided the final saliva sample (Assessment 6).

In block IV of the experiment, participants were debriefed and thanked. They were also offered snacks and water, and participants from Saint Petersburg were financially rewarded for participating in the study (~20 Euro). In addition, the experimenters observed the participants’ behaviour before they left the lab. Specifically, the experimenters marked whether participants engaged in post-experiment conversation, exchanged contact information, bid farewell to one another, or left together.

### Measurements

The methods used to generate the dataset are presented below. Variable view file contains detailed descriptions of the measures and coding for variables in the dataset. All measures were administered in Russian. English and Russian versions of the tasks and measures used can be found in the Variable view file.

#### Screening survey

A socio-demographic and screening survey was administered prior to the experiment and included questions about participants’ age, binary gender, medications, and medical conditions (e.g., infectious diseases and hormonal disorders, and pregnancy). In addition, it contained the Zoom Exhaustion & Fatigue Scale (15 items)^27^, a battery of questions on attitudes towards virtual meetings (8 items)^28^ and the Appraisal of Social Concerns questionnaire^29^ (assessed as a trait, 20 items).

Russian and English versions of all survey items, as well as their coding in the dataset, can be found in the Variable view file. Reliability estimates (Cronbach’s α) are available in Table 3 and the Variable view, Screening_processed tab. The reliability of these scales in the current study varied from satisfactory (0.64) to excellent (0.92).

#### Experimental part

##### Block I

Participants completed Russian-language versions of the Close Relationship Experience^30,31^ scale and the Big Five Inventory-2^32,33^, along with the Appraisal of social concerns^29^ scale, which was administered with modified instructions to specifically assess situational (rather than trait) social anxiety. We also collected information on participants’ age and gender, and females were asked to report their current day of the menstrual cycle.

Russian and English versions of all survey items, as well as their coding in the dataset can be found in the Variable view file. Reliability estimates (Cronbach’s α) are available in Table 3 and the Variable view, Before communication_processed tab. The reliability of these scales in the current study was good (0.83) to excellent (0.91).

##### Block II (Collaborative tasks)

**NASA Exercise: survival on the moon**. The NASA: Survival on the Moon Task^34^ required participants to negotiate a joint solution for a proposed problem. Participants were given 18 minutes to complete the task. English and Russian versions of the problem scenario are available in the Variable view file.

The task was divided into three steps.

On Step 1 participants were asked to work individually for five minutes to fill the table based on their personal opinion.

On the Step 2 participants were instructed to discuss and agree on the importance of each item with the partner within 10 minutes.

Finally, participants had three minutes to submit their personal opinions again. Participants were notified that their new opinions may differ from their joint opinion and their previous opinions. All written output of the joint task was collected and included in the dataset.

**Technical break task**. The second task was a ‘technical break’ — an artificially modelled situation of an unstructured break to observe communication without a specific task. Participants were presented with the following text: “For the next task, you will need a blank piece of white paper and a set of colored pencils”. The participants checked their desks for all the materials and found that there were no pencils. The experimenter apologised and said that they would bring pencils in a minute. Five minutes were allotted for this phase of the experiment. After the experimenter would bring the pencils, the experiment would continue.

**Creative drawing task**. The third task asked participants to draw a fictitious animal on paper using pencils. The instruction to the task was as following: “You are preparing an April Fools’ Day prank in the form of Wikipedia articles about an animal that does not exist. Your goal: to invent your own animal and help each other create its description, history and name.”

Participants were given 5 minutes to draw their animals. They were then asked to show their drawings to each other and discuss each animal for 5 minutes. The author of the drawing first listened to the ideas about their animal and then commented.

Finally, the participants had 3 minutes to come up with appropriate names for both animals. The total duration of the task thus was 18 minutes.

##### Block III

Block III of the experiment included filling out several questionnaires to assess participants’ perceived quality of contact with their communication partners. The following questionnaires were used: Interpersonal Communication Satisfaction Inventory^35^, The Networked Minds Social Presence Inventory^36^, Attraction Scale Interpersonal^37^, and Attributional Confidence Scale (CL7)^38^. In addition, participants complete the Big Five Inventory-2^32,33^ on behalf of their partners (trying to predict the partners’ answers). Russian and English versions of all questionnaires items, as well as their coding in the dataset and reliability estimates (Cronbach’s α), can be found in Table 3 and the Variable view file. The reliability of the scales in the current study was good (0.74) to excellent (0.91).

##### Block IV

During Block IV (debriefing), experimenters coded dyads’ natural behaviours (e.g., farewells, contact-sharing, exiting together) for later inclusion in observational data. Detailed information about coded behavior is provided in the Variable view file, Sample tab.

#### Repeated measurements

The measurements below were applied more than once during the experiment.

##### Positive affect and negative affect schedule

To assess participants’ emotional states, we administered the Russian version^39^ of the Positive and Negative Affect Schedule (PANAS)^40^ six times during the experiment. The Variable view file, PANAS tab provides the questionnaire items in Russian and English, their dataset coding, and reliability estimates (Cronbach’s α). In this study, reliability varied across measurements, with positive affect ranging from α = 0.88 to 0.93 and negative affect from α = 0.74 to 0.85 (Table 3).

##### Saliva collection and processing

Participant provided saliva samples by drooling into a sterile 5 ml tube six times during the experiment along with PANAS measure. Saliva samples were stored on ice throughout the experimental session and frozen immediately after the session ended. After full sample collection saliva was thawed, centrifuged for 5 min × 10000 g and supernatant was used for analysis. Oxytocin levels were quantified using a commercial enzyme-linked immunosorbent assay (ELISA) kit (Cloud-Clone Corp.). Following the manufacturer’s protocol, 50 µL of saliva was processed by sequentially adding kit reagents. Optical density was measured at 450 nm in a 96-well plate using a CLARIOstar Plus multimodal microplate reader (BMG LABTECH, Germany). Intra-assay Precision (within-assay variability) was assessed by testing three samples representing low, medium and high OXT (oxytocin) levels 20 times on the same plate, respectively. Inter-assay Precision (between-assay variability) was evaluated using the same three sample types tested across three different plates, with 8 replicated per plate. The coefficient of variation (CV) for intra-assay precision was less than 10%, and for inter-assay precision – less than 12%, meeting widely accepted ELISA performance standards. All controls and samples were analyzed in duplicate. No significant cross-reactivity or interference with OXT analogues was observed.

##### Perceptual vigilance task

A PEBL2 (version 2.1) implementation^41^ of sustained attention Perceptual Vigilance Task (PPVT) was used to assess participants’ attention and alertness before and after communication. Each trial began with a fixation cross presented for 100 ms. A red circle then appeared after a random time interval for 100 ms. Participants were instructed to press a spacebar on a keyboard as quickly as possible when they saw the red circle. Due to the random interstimulus intervals (ranging from 1000.81 ms to 9999.93 ms, mean = 5539.06 ms), the number of trials completed within the fixed 5-minute testing period varied across participants (65–121 trials; mean = 75). The Variable view file, PPVT tab describes the variables associated with this task.

##### Audio-visual recordings

Video recordings of communication during the experiment were collected using a range of consumer-grade recording devices. The specific devices include: Panasonic HC-V770 (Full HD video (1920 × 1080) at 25 frames per second in H.264 format); Sony Handycam DCR-SX85 (Standard-definition video (720 × 480) at 25 frames per second in MPEG-2 format); Xiaomi Poco M3 smartphone (Full HD video (1920 × 1080) at 30 frames per second in H.264 format.); Apple iPhone 13 Pro Max (4 K video (3840 × 2160) at 30 frames per second in H.264 format). Further, Zoom session video recordings were collected via Zoom software (client versions ranged approximately from 5.15× to 6.0×, consistent with the official release cycle) via in-built web-cameras on laptops. All recordings were made using default settings for video and audio quality.

### Ethics statement

The study protocol was approved by the Institutional Review Boards at the HSE University and Krasnoyarsk State Medical University named after Prof. V.F. Voino-Yasenetsky, and conducted according to their guidelines and regulations. Subsequently, a specialised institutional department, Information Security Centre at the HSE University, approved the sharing of data. Furthermore, The Federal Service for Supervision of Communications, Information Technology and Mass Media (Roskomnadzor) has approved cross-border data sharing. Participants were informed about the study details, including the procedures, and provided their written informed consent to participate in the research. They agreed to be recorded during the study and consented to the use of biological samples (saliva) to the extent necessary for the research project. Further, additional written consent on public access to anonymised data and controlled access to video recordings was obtained from the participants.

## Data Records

**Fig. 2:**
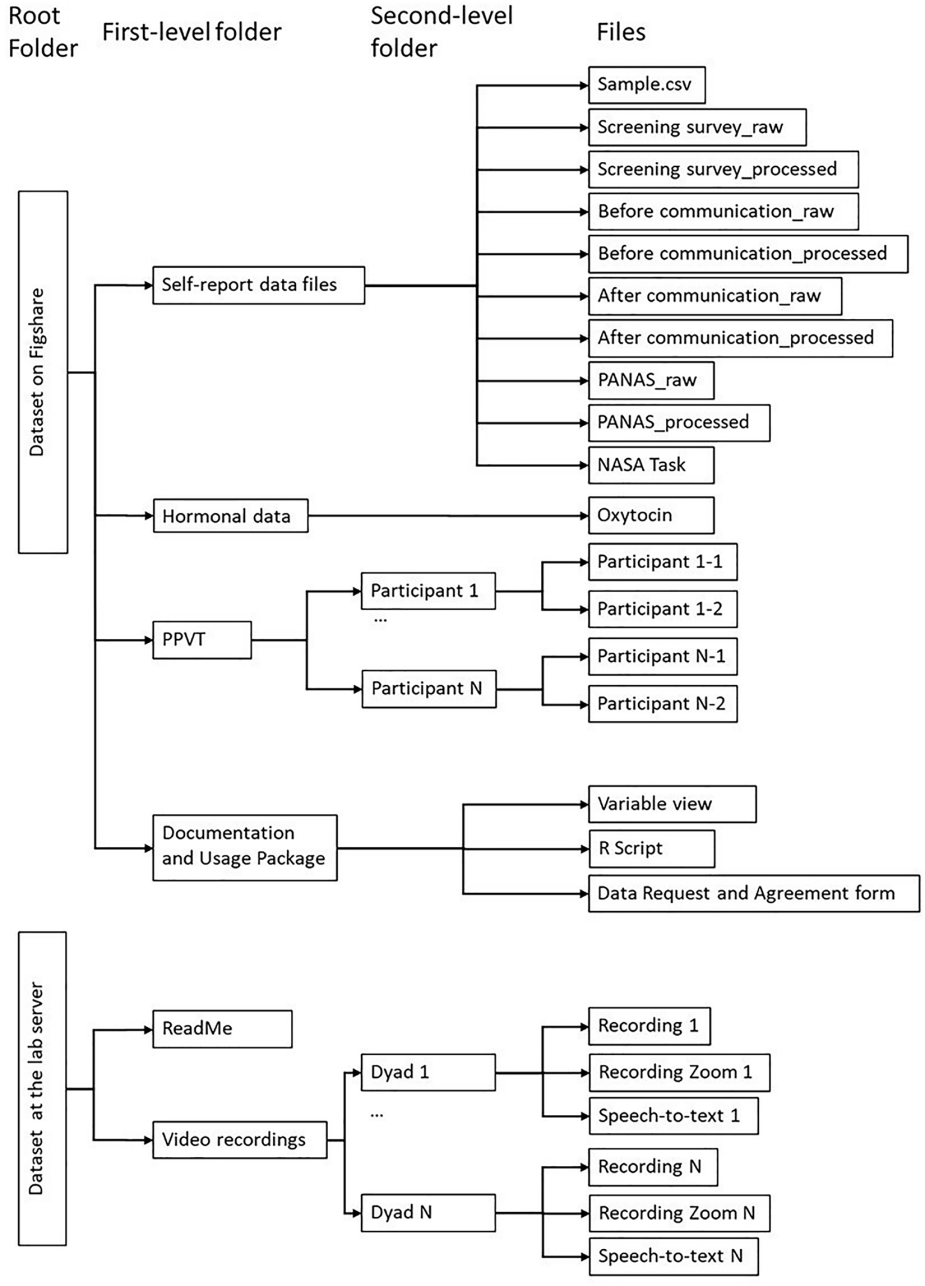
The organisation of the dataset within Figshare repository.

The anonymised part of the dataset is available on Figshare^42^ and accompanied by a Readme.txt file with the folder description. Figure 2 shows the dataset organisation. The records include raw questionnaire results on subjective measures, as well as NASA and PPVT performance and oxytocin values from 131 participants. Most of this data, including survey, questionnaire and test results, is stored in CSV format for compatibility with a wide range of data analysis packages. The folder also contains a Variable view file with detailed variable description.

Audio and video recordings and speech-to-text transcripts from 110 participants are sensitive human information that cannot be anonymised and are not publicly available due to human privacy considerations and legal restrictions. Instead, they are stored confidentially within the server of the Laboratory for Social and Cognitive Informatics (SCILa) at HSE university but can be accessed via a formal data request and usage agreement sent to SCILa email address linis-spb@hse.ru. The form for data request and usage agreement is available within the Figshare repository. Audio and video recordings are stored in MP4 format, and speech-to-text transcripts are stored in DOCX format with the ID of the speaker for each utterance.

### FigShare folder details

As it can be seen from Fig. 2, the folder on Figshare contains five subfolders that structure the data. The *Self-reported data Files* subfolder contains the *Sample.csv* file with basic information about each participant and the files corresponding to the study phases or experimental blocks (e.g., Screening survey, Before communication, After communication). Each questionnaire data file is available in two formats: raw (retaining Russian-language response labels) and processed (converted to numerical values with calculated subscale scores using R script). A file with NASA task output and PANAS results for all measurements is also placed in this subfolder. The *Hormonal data* folder contains the table with oxytocin measurement results. PPVT folder consists of subfolders titled by participant identification numbers, each of them containing two lower-level folders corresponding to two PPVT measurements. The *Documentation and Usage Package* subfolder contains the *Variable view* file with a detailed description of the questionnaires used in the experiment, the Rstudio script used to calculate the questionnaire subscales, and the *Data Request and Agreement* form.

#### Sample.csv file

This table encompasses the list of 131 participants’ IDs with corresponding Dyad IDs. It also contains information about data collection city (Saint-Petersburg or Krasnoyarsk), the date of the experimental session, and the experimental condition (online or offline). Additionally, this table presents results from post-experiment behavioural observations, coded event-by-event using predefined categories across four dimensions: (1) whether participants exited the lab together (yes/no), (2) whether they exchanged contact information (yes/no), (3) whether they engaged in conversation (yes/no), and (4) their farewell behaviuor, classified into one of four mutually exclusive combinations: no talk, no goodbye; no talk, goodbye; talk, no goodbye; or talk, goodbye. Observational data were collected for 106 participants; for the remaining individuals, observations were not conducted due to time constraints.

#### Screening_raw.csv and Screening_processed.csv files

These tables contain demographic characteristics, attitude to virtual meetings and social anxiety as a trait for 130 participants of the experiment. Data from one participant were lost due to a technical issue (an internet disconnection during online form submission). Additionally, one participant missed the VMAI questionnaire and did not provide a response. *Screening_raw.csv* file contains raw data with Russian language labels, and *Screening_processed.csv* file contains numeric data with calculated subscales.

#### Before communication_raw.csv and Before communication_processed.csv files

These files contain data on questionnaires which participants filled in before they were involved in communication with their partner (Block I) for all 131 participants, except for the Close Relationship Experience questionnaire, where a coding error in the response options affected 2 participants. *Before communication_raw.csv* file contains raw data with Russian language labels, and *Before communication_processed.csv* contains data with calculated subscales.

#### After communication_raw.csv and After communication_processed.csv files

These files contain data on questionnaires which participants filled in after they were involved in communication with their partner (Block III) from 130 participants. Data from one participant were lost due to technical issues (an internet disconnection during online form submission). *After communication_raw.csv* contains raw data with Russian language labels, and *After communication_processed.csv* file contains data with calculated subscales.

#### PANAS_raw.csv and PANAS_processed.csv files

These files contain data on the positive and negative affect schedule, which participants filled in six times during the experiment. This data is available for 125–131 participants, depending on the measurement point. Specifically, 125 observations available for measurements I-II; 130 observations for measurements III-V and full 131 – for the last measurement. The missing data were lost due to technical issues (an internet disconnection during online form submission). *PANAS_raw.csv* contains raw data with Russian language labels, and *PANAS_processed.csv* file contains data with calculated subscales.

#### NASA Task.csv

The file includes NASA task responses from 128 participants for each stage of the task: individual item rankings before discussion, collaborative decision during discussion, and individual decision after discussion. Data from three participants were excluded due to invalid responses; specifically, identical ratings assigned to multiple items, suggesting inattentive task completion and making final score calculation unreliable. As a result, the NASA Task .csv file contains three scores per participant: two unique scores (Before, After) and one score (Together) shared with their partner.

#### Oxytocin.csv

This file encompasses saliva oxytocin values for each time point (I-VI) during the experiment expressed in pg/ml. The data is available for 126–128 participants depending on measurement point. Specifically, 128 observations available for measurements I-II; 127 observations for measurements III, V and VI; 126 observations for measurement IV. Data from the remaining time points were unavailable because oxytocin could not be extracted from the submitted saliva samples due to insufficient sample volume.

#### PPVT folder, ppvt-XXXX_1.csv and ppvt-report-XXXX_1.txt

PPVT folder encompasses output files from PEBL2 software on participants’ performance on a sustained attention Perceptual Vigilance Task. PEBL2 PPVT outputs consist of two files: 1) a .csv data file containing features for each trial; 2) a .txt report file summarising the results. Each .csv and .txt file is named using the participant’s four-digit ID followed by a number indicating the measurement session (1 for before communication, 2 for after communication); the same principle is used in subfolder names. Overall, this data is available for 13265 trials (88 participants before and 88 participants after communication). The remaining data are missing due to technical issues (PEBL2 failed to generate and save the output files).

#### Variable view file.csv

Variable details are available in the *Documentation and Usage Package folder* under the *Variable view* file. This file contains tabs matching each data file, with columns labelled according to their corresponding variables. Each variable entry includes: description, item formulation in English and Russian, value labels, and scale information.

### Audio-video recordings on the lab server

Video recordings of communication during the experiment are stored in MP4 format in a repository on the laboratory server due to the sensitive nature of the data and legislation restrictions. Video recordings are stored in folders corresponding to the dyads’ IDs within the root directory. There are two types of video files: (1) recordings from side cameras positioned approximately 1 meter from each participant (available for 100 participants); (2) Zoom recordings (labelled “Zoom”) capturing the communication sessions in both conditions (available for 51 dyads). In the zoom recordings, participants appear in small on-screen windows, as captured from their frontal laptop cameras. The remaining videos are unavailable due to either technical issues, such as corrupted recordings or failure to save files owing to their large size, or participants’ non-consent to share video recordings. Each folder also contains files with Russian language speech-to-text transcripts of each dyad’s communication.

## Technical Validation

The experimental study employed a multicenter design. Project-specific personnel underwent training in accordance with standardised guidelines to guarantee consistency of the data collection process. Blinded trained experimenters conducted the experimental procedures. Furthermore, to ensure the reliability and objectivity of the data produced, random group allocation was implemented using a random number generator.

To guarantee the uniformity of the sample, the coordinator verified the eligibility of the participants following the screening survey. The exclusion criteria contained infectious and hormonal diseases, the use of hormonal medication, pregnancy, and age below 18 or above 25. Only those participants who met the eligibility criteria were invited to participate. Additional precautions were taken to ensure that participants in dyads were unfamiliar with each other prior to the experiment. Each participant was offered photographs of their potential dyad partners until both participants in the dyad confirmed that they were not acquainted. Additionally, participants were asked to arrive at the laboratory at different times with a 10-minute interval to minimise the likelihood of encountering each other before the experiment.

Since some data collection methods had not been used in Russian language before, a pilot study was carried out to validate the Russian-language versions of questionnaires. The study included 193 participants (150 females) from the same age group (*mean age* = 20.64, *SD* = 4.59) as those in the main experimental study. Measurements performed by scales/questionnaires were validated for internal reliability (i.e., Cronbach’s α, mean, IQR). All the scales showed acceptable to excellent reliability (Table 2).

**Table 2:** Reliability pre-test of adapted instruments.

Variable	Mean	SD	Min	Max	IQR	Cronbach’s α
ZEFS General Fatigue	6.27	3	0	15	4	0.91
ZEFS Visual Fatigue	5.04	2.53	0	15	3	0.84
ZEFS Social Fatigue	5.66	2.9	0	15	4	0.82
ZEFS Motivational Fatigue	5.94	3.19	0	15	5	0.87
ZEFS Emotional Fatigue	5.31	2.96	0	15	4	0.89
ZEFS total	28.22	12.12	0	75	15	0.92
VMAI Total	25.09	7.479	1	45	10	0.65
ASC trait	44.59	19.08	0	98	26	0.95
ICSI Total	62.66	21.6	0	96	27	0.93
NMSPI Attentional Allocation	23.41	9.43	0	36	12	0.92
NMSPI Message Understanding	25.62	8.89	0	36	8	0.93
NMSPI Affective Understanding	19.97	10.21	0	36	17	0.89
NMSPI Emotional Interdependence	18.44	9.33	0	36	13	0.87
NMSPI Behavioural Interdependence	20.01	9.2	0	36	12	0.86
ASI Social	19.1	8.79	0	30	12	0.88
ASI Task	18.56	8.79	0	30	12	0.87
CL7 total	46.35	14.35	6	70	22	0.94

Further, these measurements were also tested for reliability in the experiment (Table 3).

**Table 3:** Descriptive statistics and reliability of measurements.

Variable	Mean	SD	Min	Max	IQR	Cronbach’s α
OXT I	27.40	12.50	8.10	64.59	16.73	
OXT II	24.17	12.15	5.5	99.68	12.35	
OXT III	23.41	9.12	9.43	50.10	12	
OXT IV	24.26	11.19	7.20	81.19	12.78	
OXT V	23.48	10.95	3.01	76.33	13.28	
OXT VI	25.39	15.5	6.40	118.08	12.54	
PANAS I PA	31.75	6.48	16	48	8	0.88
PANAS II PA	30.82	7.56	10	50	10	0.92
PANAS III PA	32.12	7.05	16	50	9.5	0.92
PANAS IV PA	31.34	8.01	12	50	11	0.93
PANAS V PA	32.35	7.07	10	50	8.75	0.91
PANAS VI PA	28.53	8.19	10	50	10.5	0.92
PANAS I NA	14.04	4.75	10	42	5	0.86
PANAS II NA	12.4	2.99	10	21	4	0.75
PANAS III NA	12.04	2.97	10	26	3	0.8
PANAS IV NA	12.11	3.4	10	25	3	0.84
PANAS V NA	11.55	3.06	10	28	2	0.86
PANAS VI NA	12.15	3.66	10	31	2.5	0.83
ZEFS General Fatigue	6.46	2.23	0	12	3	0.81
ZEFS Visual Fatigue	4.13	1.55	0	9	2	0.7
ZEFS Social Fatigue	6.66	2.66	0	13	3	0.78
ZEFS Motivational Fatigue	6.71	2.89	0	15	5	0.82
ZEFS Emotional Fatigue	5.14	2.09	0	11	3.75	0.77
ZEFS total	29.1	9.08	0	49	13.75	0.9
VMAI Total	33.95	7.57	13	50	10	0.64
ASC trait	41.91	13.71	20	78	17	0.92
Attachment anxiety	46.3	12.84	0	72	19	0.83
Attachment avoidance	40.74	12.75	0	75	16.5	0.84
Extraversion	37.78	8.44	19	58	12	0.86
Agreeableness	44.4	7.28	22	58	11	0.82
Conscientiousness	46.31	8.46	24	65	11	0.87
Neuroticism	33.69	9	12	55	12	0.85
Openness	45.55	7.38	22	60	10	0.84
ASC state	32.75	9.95	20	77	10.5	0.91
ICSI Total	90.7	13.33	54	111	17	0.9
NMSPI Attentional Allocation	32.65	6.73	18	42	10	0.86
NMSPI Message Understanding	37.23	4.43	22	42	7	0.83
NMSPI Affective Understanding	27.34	5.7	11	42	7	0.8
NMSPI Emotional Interdependence	25.79	6.54	6	40	8	0.74
NMSPI Behavioural Interdependence	31.05	5.23	12	42	6	0.77
ASI Social	24.04	6.62	6	35	9	0.87
ASI Task	28.38	4.91	10	35	6.75	0.81
CL7 total	28.21	12.42	1	56	17.75	0.86
Extraversion Other	38.58	9.38	14	58	14	0.91
Agreeableness Other	47.56	6.4	24	60	8	0.87
Conscientiousness Other	48.22	7.581	26	63	10	0.89
Neuroticism Other	29.01	5.92	14	45	7.75	0.74
Openness Other	44.05	7.24	16	60	9	0.85

**Fig. 3:**
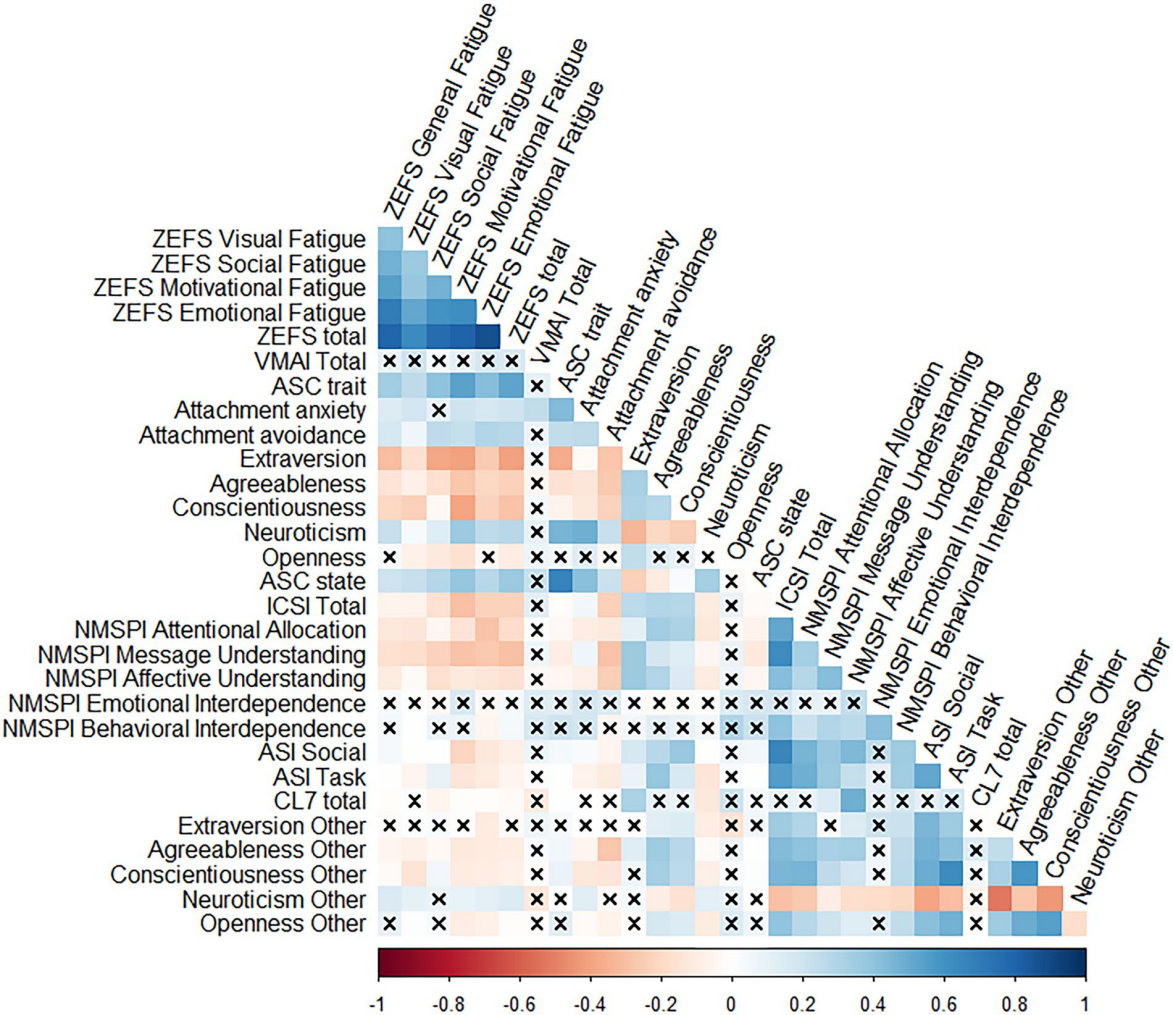
A heatmap of correlations between subjective measures in the experiment.

In addition to high reliability, high convergent validity was found for these measures, as is seen from the strong positive correlations between homogeneous tests (Fig. 3). For example, expected moderate to strong positive correlations were found for the Interpersonal Communication Satisfaction Inventory with three subscales of the Networked Minds Social Presence Inventory (*r* = .4 - .6) and with the Attraction scale (both Social and Task, *r* = .5 - .6). The measure of Appraisal of social concerns (both Trait and State) showed significant correlations with Zoom fatigue subscales (*r* = 0.2 - 0.5), Attachment Anxiety, Avoidance (*r* = .2 - .3) and Neuroticism (*r* = 0.3 - 0.5).

The null correlations observed between the heterogeneous measures provide confirmation of their high discriminant validity (see Fig. 3). Thus, individuals’ ratings of others’ personality traits show weak or non-significant relationships with their own Zoom fatigue or personality traits.

## Usage Notes

We have implemented comprehensive measures to protect participant privacy and uphold ethical standards within the scope of our control. All shared data have been anonymised, and sensitive materials (e.g., video recordings) require formal data use agreements. While full access to these data is provided, their use is strictly limited to scientific research which has been explicitly approved by the Federal Service for Supervision of Communications, Information Technology and Mass Media of the Russian Federation (Roskomnadzor).

Full access to the anonymised data as well as authors’ code is provided via Figshare^42^, while the audio and video data are stored on the server of the Laboratory of Social and Cognitive Informatics at HSE university, with restricted access. The dataset is available under a Data Use Agreement (DUA), with data access requirements given in the repository. Access to the data is unlikely to be denied – the request process exists to satisfy ethical requirements, ensuring potential users have been made aware of their responsibilities regarding the use of video containing people (namely, to treat it respectfully and to not misuse the data in a way that could be considered demeaning or which could embarrass the participants), and that the data is to be used only for research purposes.

Access to the dataset thus requires prospective users to apply for access via a form available on Figshare. Access is restricted to principal investigators and affiliated researchers at institutions that have signed a DUA. To protect the privacy of research participants, researchers interested in accessing the dataset must first fill in the DUA. Once completed, the form should be submitted to the laboratory email linis-spb@hse.ru. Approval decisions will be communicated within two weeks; however, unforeseen delays may occur if additional information is required.

This dataset provides behavioural and physiological measurements from a controlled dyadic interaction experiment, designed for research in social neuroscience and psychology. It includes: 188 anonymized CSV files with psychological and physiological data (e.g., saliva oxytocin, psychological scales, attention test) and audio/video recordings.

## Data Availability

The anonymised dataset (questionnaire results, NASA/PPVT performance, oxytocin values) is archived on Figshare^42^ (10.6084/m9.figshare.29545964). Sensitive audio/video recordings and speech-to-text transcripts are stored confidentially at the Laboratory for Social and Cognitive Informatics (SCILa), HSE University, and available upon request to linis-spb@hse.ru; the data request form is included in the Figshare repository.

## Code availability

The current dataset contains raw data in standard formats that can be accessed and analysed using standard software and libraries. Raw CSV files with Cyrillic text should be opened with CP1251 (Windows Cyrillic) encoding, e.g., in R: read.csv(“file.csv”, fileEncoding = “CP1251”). To facilitate processing and reproducibility, we have included a custom R Markdown script (R version 4.3.3 (2024-02-29 ucrt), RStudio 2025.5.0.496), which is available in the same Figshare repository as the data. No additional pre-processing pipelines for audio/video data were developed.

## References

[1] Ignatowicz, A. *et al*. Internet videoconferencing for patient–clinician consultations in long-term conditions: A review of reviews and applications in line with guidelines and recommendations. *Digital health* **5**, 2055207619845831 (2019). 31069105 10.1177/2055207619845831 PMC6495459

[2] Thomas, N. *et al*. Review of the current empirical literature on using videoconferencing to deliver individual psychotherapies to adults with mental health problems. *Psychology and Psychotherapy: Theory, Research and Practice* **94**, 854–883 (2021). 10.1111/papt.12332 PMC8451850 33620133

[3] Adipat, S. Why Web-Conferencing Matters: Rescuing Education in the Time of COVID-19 Pandemic Crisis. *Frontiers in Education* **6** (2021).

[4] Sourdin, T., Li, B. & McNamara, D. M. Court innovations and access to justice in times of crisis. *Health Policy and Technology* **9**, 447–453 (2020). 32895624 10.1016/j.hlpt.2020.08.020 PMC7456584

[5] Basch, J. M., Melchers, K. G., Kurz, A., Krieger, M. & Miller, L. It Takes More Than a Good Camera: Which Factors Contribute to Differences Between Face-to-Face Interviews and Videoconference Interviews Regarding Performance Ratings and Interviewee Perceptions? *J Bus Psychol* **36**, 921–940 (2021). 32929301 10.1007/s10869-020-09714-3 PMC7482058

[6] Archibald, M. M., Ambagtsheer, R. C., Casey, M. G. & Lawless, M. Using Zoom Videoconferencing for Qualitative Data Collection: Perceptions and Experiences of Researchers and Participants. *International Journal of Qualitative Methods* **18**, 1609406919874596 (2019).

[7] Short, J., Williams, E. & Christie, B. *The Social Psychology of Telecommunications*. (Wiley, London; New York, 1976).

[8] Daft, R. L. & Lengel, R. H. Organizational Information Requirements, Media Richness and Structural Design. *Management Science* **32**, 554–571 (1986).

[9] Reicher, S. D., Spears, R. & Postmes, T. A Social Identity Model of Deindividuation Phenomena. *European Review of Social Psychology* **6**, 161–198 (1995).

[10] Walther, J. B. Interpersonal Effects in Computer-Mediated Interaction: A Relational Perspective. *Communication Research* **19**, 52–90 (1992).

[11] Hantula, D. A., Kock, N., D’Arcy, J. P. & DeRosa, D. M. Media Compensation Theory: A Darwinian Perspective on Adaptation to Electronic Communication and Collaboration. In *Evolutionary Psychology in the Business Sciences* (ed. Saad, G.) 339–363, 10.1007/978-3-540-92784-6_13 (Springer Berlin Heidelberg, Berlin, Heidelberg, 2011).

[12] Walther, J. B. Computer-Mediated Communication: Impersonal, Interpersonal, and Hyperpersonal Interaction. *Communication Research* **23**, 3–43 (1996).

[13] Tsigeman, E., Mararitsa, L., Gundelah, O., Lopatina, O. & Koltsova, O. Psychological Aspects of Face-To-Face Versus Computer-Mediated Interpersonal Communication: An Integrative Review. in *Social Computing and Social Media* (eds Coman, A. & Vasilache, S.) **vol. 14705** 29–48, (Springer Nature Switzerland, Cham, 2024).

[14] Brucks, M. S. & Levav, J. Virtual communication curbs creative idea generation. *Nature* **605**, 108–112 (2022). 35477754 10.1038/s41586-022-04643-y

[15] Boland, J. E., Fonseca, P., Mermelstein, I. & Williamson, M. Zoom disrupts the rhythm of conversation. *Journal of Experimental Psychology: General* **151**, 1272–1282 (2022). 34748361 10.1037/xge0001150

[16] Schwartz, L. *et al*. Technologically-assisted communication attenuates inter-brain synchrony. *NeuroImage* **264**, 119677 (2022). 36244598 10.1016/j.neuroimage.2022.119677

[17] Sears, G., Zhang, H., Wiesner, W., Hackett, R. & Yuan, Y. A comparative assessment of videoconference and face-to-face employment interviews. *Management Decision* **51** (2013).

[18] Riedl, R., Kostoglou, K., Wriessnegger, S. C. & Müller-Putz, G. R. Videoconference fatigue from a neurophysiological perspective: experimental evidence based on electroencephalography (EEG) and electrocardiography (ECG). *Sci Rep* **13**, 18371 (2023). 37884593 10.1038/s41598-023-45374-y PMC10603122

[19] Balters, S., Miller, J. G., Li, R., Hawthorne, G. & Reiss, A. L. Virtual (Zoom) Interactions Alter Conversational Behavior and Interbrain Coherence. *J. Neurosci.* **43**, 2568–2578 (2023). 36868852 10.1523/JNEUROSCI.1401-22.2023 PMC10082458

[20] Kossaifi, J. *et al*. SEWA DB: A Rich Database for Audio-Visual Emotion and Sentiment Research in the Wild. *IEEE Trans. Pattern Anal. Mach. Intell.* **43**, 1022–1040 (2021). 31581074 10.1109/TPAMI.2019.2944808

[21] Cafaro, A. *et al*. The NoXi database: multimodal recordings of mediated novice-expert interactions. in *Proceedings of the 19th ACM International Conference on Multimodal Interaction* 350–359, 10.1145/3136755.3136780 (ACM, Glasgow UK, 2017).

[22] Gu, Y. *et al*. The ECOLANG Multimodal Corpus of adult-child and adult-adult Language. *Sci Data* **12** (2025). 10.1038/s41597-025-04405-1 PMC11739475 39819961

[23] Palmero, C. *et al*. Context-Aware Personality Inference in Dyadic Scenarios: Introducing the UDIVA Dataset. in *2021 IEEE Winter Conference on Applications of Computer Vision Workshops (WACVW)*. 10.1109/wacvw52041.2021.00005 1–12 (IEEE, Waikola, HI, USA, 2021).

[24] Park, C. Y. *et al*. K-EmoCon, a multimodal sensor dataset for continuous emotion recognition in naturalistic conversations. *Sci Data* **7** (2020). 10.1038/s41597-020-00630-y PMC7479607 32901038

[25] Zang, X. *et al*. Photochat: A human-human dialogue dataset with photo sharing behavior for joint image-text modeling. In *Proceedings of the 59th Annual Meeting of the Association for Computational Linguistics and the 11th International Joint Conference on Natural Language Processing* (Volume 1: Long Papers) (pp. 6142–6152) (2021).

[26] Poria, S. *et al*. Meld: A multimodal multi-party dataset for emotion recognition in conversations. In *Proceedings of the 57th annual meeting of the associationfor computational linguistics* (pp. 527–536) (2019).

[27] Fauville, G., Luo, M., Queiroz, A. C. M., Bailenson, J. N. & Hancock, J. Zoom Exhaustion & Fatigue Scale. *Computers in Human Behavior Reports* **4**, 100119 (2021).

[28] Kuhn, K. M. The constant mirror: Self-view and attitudes to virtual meetings. *Computers in Human Behavior* **128**, 107110 (2022).

[29] Telch, M. J. *et al*. Appraisal of social concerns: A cognitive assessment instrument for social phobia. *Depression and Anxiety* **19**, 217–224 (2004). 15274170 10.1002/da.20004

[30] Казанцева, Т. В. Адаптация модифицированной методики «Опыт близких отношений» К. Бреннан и P. К. Фрейли. *Известия Российского государственного педагогического университета им. А.И. Герцена* **2**, 139–143 (2008).

[31] Brennan, K. A., Clark, C. L. & Shaver, P. R. Self-report measurement of adult attachment: An integrative overview. *Attachment theory and close relationships* **46**, 76 (1998).

[32] Калугин, А. Ю., Щебетенко, С. А., Мишкевич, А. М., Сото, К. Д. & Джон, О. Психометрика русскоязычной версии Big Five Inventory–2. *psychology* **18**, 7–33 (2021).

[33] Soto, C. J. & John, O. P. The next Big Five Inventory (BFI-2): Developing and assessing a hierarchical model with 15 facets to enhance bandwidth, fidelity, and predictive power. *Journal of Personality and Social Psychology* **113**, 117–143 (2017). 27055049 10.1037/pspp0000096

[34] Eddy, J. Survival on the Moon… or “The Nasa Game”. *Science Activities: Classroom Projects and Curriculum Ideas* **5**, 28–30 (1971).

[35] Hecht, M. The conceptualization and measurement of interpersonal communication satisfactioch. *Human Communication Research* **4** (1978).

[36] Harms, C. & Biocca, F. Internal consistency and reliability of the networked minds measure of social presence. in *Seventh annual international workshop: Presence***vol. 2004** (Universidad Politecnica de Valencia Valencia, Spain, 2004).

[37] McCroskey, J. C. & McCain, T. A. The measurement of interpersonal attraction. *Speech Monographs* **41**, 261–266 (1974).

[38] Clatterbuck, G. W. Attributional confidence and uncertainty in initial interaction. *Human Comm Res* **5**, 147–157 (1979).

[39] Осин, Е. Н. Измерение позитивных и негативных эмоций: разработка русскоязычного аналога методики Panas. *Психология. Журнал Высшей школы экономики* **9**, 91–110 (2012).

[40] Watson, D., Clark, L. A. & Tellegen, A. Development and validation of brief measures of positive and negative affect: The PANAS scales. *Journal of Personality and Social Psychology* **54**, 1063–1070 (1988). 3397865 10.1037//0022-3514.54.6.1063

[41] Dinges, D. F. & Powell, J. W. Microcomputer analyses of performance on a portable, simple visual RT task during sustained operations. *Behavior Research Methods, Instruments, & Computers* **17**, 652–655 (1985).

[42] SCILa lab. A dataset on the experimental study of online and offline communication with digital and biomarkers. 2262098 Bytes figshare 10.6084/M9.FIGSHARE.29545964 (2026). 10.1038/s41597-026-07382-1 PMC13408955 42135355

